# Tetz’s theory and law of longevity

**DOI:** 10.1007/s12064-018-0267-4

**Published:** 2018-07-05

**Authors:** George Tetz, Victor Tetz

**Affiliations:** Human Microbiology Institute, 423 West 127 Street, New York, NY 10027 USA

**Keywords:** Tetz’s law, Longevity, Aging, Individual Pangenome, Microbiome

## Abstract

Here, we present new theory and law of longevity intended to evaluate fundamental factors that control lifespan. This theory is based on the fact that genes affecting host organism longevity are represented by subpopulations: genes of host eukaryotic cells, commensal microbiota, and non-living genetic elements. Based on Tetz’s theory of longevity, we propose that lifespan and aging are defined by the accumulation of alterations over all genes of macroorganism and microbiome and the non-living genetic elements associated with them. Tetz’s law of longevity states that longevity is limited by the accumulation of alterations to the limiting value that is not compatible with life. Based on theory and law, we also propose a novel model to calculate several parameters, including the rate of aging and the remaining lifespan of individuals. We suggest that this theory and model have explanatory and predictive potential to eukaryotic organisms, allowing the influence of diseases, medication, and medical procedures to be re-examined in relation to longevity. Such estimates also provide a framework to evaluate new fundamental aspects that control aging and lifespan.

## Introduction

The quest for increasing human longevity is the subject of intense study; increase of the human lifespan by postponement of death, preservation of life via the support and maintenance of vital functions, or the achievement of a productive lifespan, for as long as possible, is the most commonly accepted goals of medicine. Consequently, a large number of theories describe lifespan as a result of the interaction of biological, social, economic, genetic, and other factors (Christensen et al. 2006; Rice and Fineman 2004). An individual’s lifespan is largely determined by aging, which is considered a dynamic process leading to the continuous adaptation of the body to lifelong exposure to a variety of stressors (Franceschi et al.^1995^). Therefore, according to Capri et al. (2006), aging represents the ways in which the organism adapts to harmful stressors by using genetic, epigenetic, and environmental influences.

The numerous models of the aging process, which are predominantly based on the forces of natural selection, free radical damage, and telomere-shortening theories, represent a highly rich and complex field of research (Gavrilov and Gavrilova 2001; Harman 1992; Johnson et al. 1999; Weinert and Timiras 2003).

One of the first aging models was the concept of the force of natural selection that was widely discussed by Fisher (1930), Haldane (1941), Medawar (1946, 1952), Williams (2001), Hamilton (1966), Charlesworth (2001) to explain the changes—also referred to as aging—that occur in adult organisms over the course of their lifetimes. The concept of the force of natural selection developed in these studies, particularly in Charlesworth’s study, explained the differences in the effects of mutations between organisms of different ages, stating that mutations exert varying and cumulative effects on organisms according to their age. It was postulated that aging is a result of processes that may be safe and favorable early in life, but exert cumulative negative impacts later on. As Charlesworth (2001) states, “A mutation might cause increased mortality at all ages after a certain age, e.g., because of the accumulation of some harmful product”.

More recently, aging has been defined as the accumulation of diverse cellular, tissue, and organ changes that are responsible for the increased risk of disease and death, thus representing the main limitation of longevity (Harman 2003).

A number of environmental factors affect the aging and longevity of individuals; these include various chemical agents (e.g., food-derived xenobiotics, polluted air, and carcinogenic microflora-derived metabolites) (Carmody and Turnbaugh 2014; Motorykin et al. 2013), physical agents [ultraviolet (UV) radiation] (Berneburg et al. 2000), and biological agents (Finch 2010). The effect of such factors predominantly involves damage to DNA via the generation of reactive oxygen and nitrogen species, alkylating agents, or other compounds, leading to the occurrence of mutations and the accumulation of macromolecular changes (Wiseman and Halliwell 1996). These changes exert major effects on the cells in which they occur, ultimately influencing the longevity of the organism as a whole (Maynard et al. 2015). In addition, endogenous factors cause changes throughout the lifetime of the organism. For example, recombination or viral transformation leads to the alteration and reconstruction of genetic material, which affects lifespan (Regan and Laimins 2013). In addition, alterations to the genome of the cells of the macroorganism may result from intrinsic mutations acquired during cell division (Stratton et al. 2009).

The longevity of organisms has been calculated using a wide range of mathematical models. The identification and measurement of markers of aging represent a predominant component of research on aging, and are widely used for the mathematical modeling and prediction of lifespan (Engelfriet et al. 2013). Because of the complexity of aging and the wide range of ways of techniques involved in the modeling and prediction of this process, over 300 theories and models are currently used for the evaluation and analysis of lifespan and longevity (Edelstein-Keshet et al. 2001; Weinert and Timiras 2003).

These models, which are based on statistics of life expectancy and mortality dynamics, or the rate of aging, often require specialized assumptions (Avraam et al. 2013). For example, Gompertz’s law is widely used for the prediction of mean longevity in population studies (Gompertz 1825; Mueller et al. 1995). This law is based on data, showing that the mortality rate of all human populations increases with age after sexual maturity, demonstrating direct geometrical progression. Therefore, according to this law, the mortality rate doubles with every 8-year increase in an adult’s age. However, existing models generally focus on the macroorganism and its cells as the main subject of longevity studies. The field of oncogenomics investigates the role of variability in mammalian genes in the development of malignancies as one of the main factors that limit lifespan. Futreal et al. (2004) suggested the “census” theory, whereby mutations in more than 1% of human genes contribute to human cancer and reduce life expectancy. At the same time, the majority of human genome is represented by a non-coding DNA sequences that do not encode protein sequence (Lander et al. 2001). Today, it is becoming obvious that not only alterations in coding DNA, but also in a non-coding DNA components are implicated in human pathologies (Shihab et al. 2015).

The Pangenome concept is an additional theory that describes the ways in which the collective genetic network of all living organisms interacts with non-living genetic elements (NLGE) involved in the storage and transmission of genetic information (Tetz 2005). This theory considers life and death from the perspective of the development of new genes and their distribution within the united genetic network. NLGE are represented by non-living objects that contain genetic information such as viruses, plasmids, transposons, and extracellular DNA and RNA.

This pool of mobile genetic elements is a complex system known to provide dynamic stability to the components of both the microbiome and macroorganism. In parallel, mammalian organisms (particularly humans) and microbial symbionts interact in a cooperative way that affects the key vital processes of the host (Jones et al. 2010).

The human microbiota includes bacteria, archaea, protozoa, fungi, and viruses (bacteriophages) in dynamic equilibrium with the host. However, viruses are sometimes excluded from the category of microbiota as they are not considered live organisms; the special term “Virobiota” is used to describe this group (Abeles and Pride 2014). Currently, approximately 1000 bacterial species are known to comprise the microbiota present in the human body, representing an overwhelming minority of the total known bacterial diversity (Rajilić-Stojanović and de Vos 2014). The collective genes of all microbes comprising the human microbiota, which is termed the “microbiome”, are 400 times larger in number than the total amount of genes in all human cells; in addition, the human body is estimated to contain a great number of bacteria whose quantity, according to different studies, varies from the widely cited 10:1 ratio (the number of bacteria exceeds the number of human cells) to the same order as the number of human cells, with about 100 times more genes overall (Rosner 2014; Sender et al. 2016; Yang et al. 2009). For example, the human genome encodes less than 30,000 protein-coding genes, whereas the microbiome is represented by 3.3 million unique genes (Claverie 2001; Qin et al. 2010). The microbiome exerts a significant influence on human health and well-being; therefore, some workers have expanded beyond the concept that the human organism is composed of solely eukaryotic cells, and consider the human body as a superorganism comprising of both microbial and human cells (Sleator 2010; Zhu et al. 2010).

Gut microbes colonize most mucosal surfaces and contribute to host metabolism. Recent studies have also shown that these microbes are involved in the development and normal function of the immune system, neural systems, and gastrointestinal tract; in addition, these microorganisms have been shown to contribute to mucosal permeability (Borre et al. 2014; Peterson and Artis 2014).

Disruption of the microbiome is implicated in numerous human disorders (Carding et al. 2015); almost all alterations in the microbiota, leading to the loss of dynamic equilibrium with the host organism, are considered harmful to the host, causing a variety of mammalian pathologies (Clemente et al. 2012; Tetz et al. 2016). Quantitative and qualitative alterations to the gastrointestinal microbiota and bacterial overgrowth in the small intestine are associated with the development of numerous diseases such as irritable bowel syndrome, inflammatory bowel disease, rheumatoid arthritis, and intestinal carcinogenesis (Gueimonde et al. 2007; Shreiner et al. 2015). Some studies have additionally shown that gut microbiota influence and participate in regulation of the lifespan, with individual longevity attributed to specific microbiota (Ottaviani et al. 2011; Portal-Celhay et al. 2012).

This article aims to introduce new ideas leading to a novel theory of longevity that describes the mechanisms underlying the limitations to lifespan, the aging process, and the causes of mortality; these mechanisms are based on the collective contribution of alterations (due to external and intrinsic factors) in the genes of both the macroorganism and its microbiome to determining the individual lifespan of eukaryotic organisms.

## Methods

Our methods are integrated into the body of the manuscript, primarily in the “Results” section.

## Results

### Tetz’s theory of longevity: general overview

Tetz’s theory of longevity is a body of ideas and a mathematical model that are intended to predict, estimate, and improve longevity, by considering the host organism consisting of several genetic subpopulations: host eukaryotic cells (including all materials in all ‘fluids’), representatives of commensal microbiota, and their NLGEs.

The theory suggests that individual longevity could be estimated by considering alterations that occur in the DNA of the host organism, including its resident microbiota and associated NLGEs, as important populations playing an important role in maintaining host health, diseases, lifespan, and aging. To develop the mathematical model based on Tetz’s theory of longevity, we proceeded from some initial points.

The totality (or sum) of DNA that affects the lifespan of an individual is the total DNA in all cells of the macroorganism, microbiome, and NLGEs. We identified NLGEs associated with the microbiota (e.g., bacteriophages, transposons, plasmids, and microbial cell-free DNA and RNA) and those associated with the host organism (e.g., viruses, plasmids, and eukaryotic cell-free nucleic acids). The totality of DNA of the macroorganism is represented by the DNA of every cell of the macroorganism and the NLGE associated with the host organism. The microbiome’s totality of DNA includes the total DNA from representatives of residential flora, including bacteria, archaea, fungi, protozoa, and NLGEs that are associated with the microbiota. Thus, we introduce the novel term “Individual Pangenome” to reflect the totality of all DNA of all cells of the macroorganism, microbiome, and their NLGEs that reflects how each component influences the lifespan of the individual, separately and cumulatively.

Tetz’s theory of longevity states that: *longevity and aging are determined by the accumulation of alterations in the totality of DNA of the host macroorganism*, *microbiota*, *and their NLGEs*.

Tetz’s theory of longevity assumes that, in an ideal theoretical model, a macroorganism could be immortal when cooperation with microbiota is ideal, influence from the outer environment is absent, and intrinsic mutations of the Individual Pangenome are absent. Aging of the host organism is the incidence and accumulation of alterations in the Individual Pangenome. Thus, the lifespan of the individual represents a process of incidence and accumulation of alterations in the Individual Pangenome, while reaching the limit of these alterations is not compatible with life.

Based on this interpretation, we propose Tetz’s law of longevity as depicted by Eq. (3). The law states that longevity is limited by the accumulation of the limiting value of alterations in the Individual Pangenome. Tetz’s law defines the lifespan of a macroorganism as: *lifespan is the time required for the accumulation of alterations in the Individual Pangenome to the limiting value that is not compatible with life*. In other words, the lifespan is the withdrawal of the maximum capacity of alterations in the Individual Pangenome that are compatible with life. Under these assumptions, the Individual Pangenome may be modeled as the totality of DNA of the host macroorganism and associated microbiota. Let us use *i* as the total number of DNA in a macroorganism and NLGEs that are associated with the host organism and *j* as the total number of DNA in associated microbiota and NLGEs that are associated with microbiota. Then, the Individual Pangenome is represented by vectors *a *= (*a*_1_, _…_, *a*_*i*_) и *b *= (*b*_1_, _…_, *b*_*j*_), where a_i_ is the DNA of macroorganism and NLGEs associated with the host organism, and b_j_ is the DNA of the microbiota and NLGEs that are associated with microbiota. The Individual Pangenome is written as follows:

1 $$IP = a + b.$$

Based on this definition of the Individual Pangenome, alterations to the Individual Pangenome are equal to the sum of all of the qualitative and quantitative alterations in the totality of DNA of the macroorganism, microbiota, and associated NLGEs. Therefore, the total number of alterations in the Individual Pangenome is as follows:

2 $$q = q_{1} + q_{2} ,$$

where *q*_1_ is the totality of qualitative and quantitative alterations in the DNA of the macroorganism and NGLEs that are associated with the host organism, and *q*_2_ is the totality of qualitative and quantitative alterations in the DNA of the microbiota and NLGEs that are associated with the microbiota. We define *q*_1_ as a value that reflects the totality of qualitative and quantitative alterations across all DNA of the macroorganism and associated NLGEs compared to an indicator of the “ideal” estimate of the qualitative and quantitative composition of the total DNA in the macroorganism. This provides ideal cooperation with the microbiota and biological immortality in the absence of any external influence or intrinsic mutations.

Qualitative alterations reflect nucleotide sequence variations that emerge in totality of all DNA sequences of the macroorganism. It is obvious that various alterations in coding and non-coding DNA components and in different genes impact host longevity differently (Shihab et al. 2015). For example, the probability that somatic mutations that are acquired during a person’s lifetime in *TP*53 or *BRAF* genes increase the risk of developing malignancy and reducing lifespan is much higher than a mutation in non-coding element or in low-penetrance (low risk) cancer-susceptibility alleles or nonfunctional junk DNA or redundant genes (Davies et al. 2002; Kellis et al. 2014; Vousden and Lane 2007).

Conversely, quantitative alterations reflect quantitative alterations of DNA, including increase or decrease in the number of genes in the macroorganism because of variation in cell counts or gene amplification and deletion. Different quantitative alterations of body-cell composition that lead to an increase in the number of genes in the macroorganism influence its life expectancy differently. For example, an increase in cell number and, thus, the total number of genes as a result of benign tumor will not affect life expectancy to the same extent as an increase in cell and gene numbers in neoplastic processes, like melanoma (Helfand et al. 2001). Moreover, an increase of certain types of NLGE (such as cell-free DNA) or the overexpression of certain plasmids may promote cancer metastasis (Aarthy et al. 2015; Lv et al. 2012).

Thus, *q*_1_ reflects both quantitative and qualitative alterations that occur in the total sum of an organism’s DNA of all cells in a macroorganism and associated NLGEs, reflecting their significance on macroorganism longevity. As in the case of alterations to DNA in macroorganism, we assume that the same consistent patterns would be present in an impact assessment of alterations to the microbiome on host longevity. Thus, we suggest that all alterations in the composition of microbiota that are expressed by *q*_2_ affect the lifespan of the host.

The main limitation when evaluating the influence of alterations to a microbiome on the longevity of the host organism is the lack of studies on its composition and the absence of acceptable determinants for the normal range of the microbiome (i.e., there is no normal range for microbiome composition) (Human Microbiome Project Consortium 2012). Tetz’s theory of longevity states that the macroorganism can be immortal under certain ideal conditions; thus, we defined normal range as a totality of DNA in all representatives of microbiota and associated NLGEs that provide ideal cooperation with the macroorganism at this time point and immortality to the host, assuming the absence of any influence from the outer environment and the absence of intrinsic mutations. Thus, *q*_2_ indicates both quantitative and qualitative changes that happen in the microbiome and associated NLGEs compared with the state of ideal cooperation with the macroorganism where they provide immortality.

Qualitative alterations to the microbiome reflect nucleotide sequence variations that emerge in DNA of all representative microbiota and NLGEs associated with microbiota. However, as in the case of alterations to host cells, not all alterations in microbiota affect the lifespan of the macroorganism in the same way. For example, reduced endopeptidase activity in *Escherichia coli* as a result of mutation will not affect human lifespan as much as the activation of enzymes leading to the increase of the amount of cancer-promoting metabolites (Schwabe and Jobin 2013). So far, we assumed that the lifespan of the macroorganism is affected by all quantitative changes that lead to alterations in the DNA in the microbiome of bacteria, archaea, fungi, and protozoa. Quantitative alterations in the microbiome reflect an increase or decrease in the total sum of DNA from the microbiome primarily because of variation in microorganism counts. Such variation may arise due to an increase or decrease in any population and the appearance of new species or disappearance of other species. However, different changes lead to different consequences to the host. For example, a moderate shift in the number of *E. coli* will not have major consequences on host longevity. In contrast, an increase in the number of *Bacteroides fragilis* in gut microbiota will negatively affect host longevity because of its association with colon carcinogenesis (Schwabe and Jobin 2013; Sears et al. 2014; Zhang et al. 2015). NLGEs associated with microbiota also influence the equilibrium of microflora and, thus, affect how microflora interacts with hosts. For example, the acquisition of certain bacteriophages or alterations to cell-free DNA lead to significant shifts in microbiota composition (Kåhrström 2015; Tetz et al. 2009). Thus, *q*_2_ reflects both quantitative and qualitative alterations that emergence in microbiota and associated NLGEs, representing their significance on the longevity of the macroorganism.

### Equation of Tetz’ law and lifespan

The limiting value of alterations in the Individual Pangenome that lead to the death of the host organism may be defined as the sum of all qualitative and quantitative alterations in the totality of DNA of the macroorganism and associated microbiota and their NGLEs that reach the limiting value. Let us denote *q*^sup^ as the limiting value (or extremum) of alterations in the Individual Pangenome that leads to the death of the host macroorganism. Therefore, conditions for the existence of the Individual Pangenome may be defined as the sum of qualitative and quantitative alterations of DNA in the macroorganism and microbiota that is below than limiting value. Then, Tetz’s law of longevity may be expressed as follows:

3 $$q_{1} + q_{2} = q\text{ < } q^{\sup } ,$$

where *q* equals the totality of qualitative and quantitative alterations in the DNA of the Individual Pangenome; *q*_1_ equals the totality of qualitative and quantitative alterations in the DNA of the macroorganism and associated NGLEs; *q*_2_ equals the totality of qualitative and quantitative alterations in the DNA of microbiota and associated NGLEs; $\forall q <$ *q*^sup^ equals the range of permissible alterations in the Individual Pangenome under which the macroorganism remains alive; $\forall q \ge$ *q*^sup^ is when the macroorganism is dead. Thus, *q*_1_ and *q*_2_ reflect both quantitative and qualitative alterations that occur in the total sum of all DNA of all cells in the macroorganism, microbiome, and their NLGEs, representing their significance on *q*^sup^ and macroorganism longevity.

Under the Tetz’ law, longevity is defined as the time [0, *t*^sup^] required for the accumulation of alterations in the Individual Pangenome to the limiting value that is not compatible with life. It is expressed as follows:

4 $$t^{\sup } :\;q_{1} \left( {t^{\sup } } \right) + q_{2} \left( {t^{\sup } } \right) = q^{\sup } ,$$

where *t*^sup^ is the time of death of the macroorganism, whereby function *q*(*t*) attains a value of *q*^sup^ for the first time. According to equation, the lifetime of the macroorganism is limited by the accumulation of alterations in the Individual Pangenome or, in other words, by the totality of alterations to DNA in the macroorganism, microbiota, and their NGLEs.

*Tetz’s law states that longevity is limited by the accumulation of alterations in the Individual Pangenome to the limiting value that is not compatible with life.*

### Tetz’s theory of longevity for evaluating the lifespan of individuals

Tetz’s theory of longevity allows the remaining lifespan and rate of aging of individuals to be calculated. The remaining lifespan is expressed in both time and the totality of qualitative and quantitative alterations to DNA in the Individual Pangenome that could occur before reaching the limiting value *q*^sup^ at *t*^sup^.

Thus, the excess (i.e., remaining possible) of maximum permissible level of alterations in the Individual Pangenome, expressed as *q*^max^, is defined as the difference between the number of limiting value of alterations in the Individual Pangenome that is not compatible with life and the value of already altered DNA in the Individual Pangenome up to the present time. Mathematically, the excess of maximum permissible level of alterations in the Individual Pangenome is represented as follows:

5 $$q^{\rm{max} } = q^{\sup } - q\left( t \right),$$

where *q*^max^ equals the excess of maximum permissible level of alterations in the Individual Pangenome.

Another function reflects alterations in the Individual Pangenome that occur at a certain time period and is expressed as follows:

6 $$\Delta q = q\left( {t_{2} } \right) - q\left( {t_{1} } \right).$$

Here, Δ*q* reflects the decrease of the excess permissible level of alterations in the Individual Pangenome at a certain time period. A negative value may be caused when the value of alterations in the Individual Pangenome at *t*_2_ exceed the excess of maximum permissible level of alterations in the Individual Pangenome at the previous time point *t*_1_. This situation occurs when there was an alteration in the Individual Pangenome at the previous time point *t*_1_ that was corrected by the time *t*_2_ was reached. This dynamic is compatible with a “hit-and-run” scenario; for instance, if a certain alteration appeared in the Individual Pangenome at *t*_1_ but was corrected at *t*_2_, other changes in the Individual Pangenome may have been triggered before *t*_2_ and not eliminated following it, resulting in their continuing to affect the lifespan of the individual (Niller et al. 2011).

To determine the decrease in the remainder of maximum permissible level of alterations in the Individual Pangenome *q*^max^(*t*) for a certain time period, the equation is expressed as follows:

7 $$q^{\rm{max} } \left( {t_{2} } \right) = q^{\rm{max} } \left( {t_{1} } \right) -\Delta q,$$

where Δ*q* is defined by Eq. (6).

Two other important characteristics are the rate of accumulation of alterations and the rate of reduction in the remainder of the maximum permissible level of alterations at a certain time point. Both are considered as aging rates, expressed as follows:

8 $$\mathop {\lim }\limits_{t \to 0} \frac{{\Delta q}}{{\Delta t}} = \frac{{{\text{d}}q}}{{{\text{d}}t}} = \frac{{{\text{d}}(q^{\sup } - q^{\rm{max} } )}}{{{\text{d}}t}} = \frac{{{\text{d}}q^{\sup } }}{{{\text{d}}t}} - \frac{{{\text{d}}(q^{\rm{max} } )}}{{{\text{d}}t}} = - \frac{{{\text{d}}(q^{\rm{max} } )}}{{{\text{d}}t}} = V(t);\quad \frac{{{\text{d}}q^{\sup } }}{{{\text{d}}t}} = 0.$$

This equation accounts for the accumulation of altered DNA in the Individual Pangenome at a certain time period, and allows the rate of change in the Individual Pangenome to be estimated by comparing this parameter at different time points. The *V*(*t*) value may also represent the rate of withdrawal of the maximum capacity of alterations in the Individual Pangenome that are compatible with life at a certain time period. The mean value of *V*(*t*) may be defined for different demographic groups allowing the normal range of *V*(*t*) to be determined. This normal criterion could then be applied to determine life expectancy based on the individual *V*(*t*) parameter values.

### Modeling the remaining lifespan of an individual

Based on Tetz’s theory of longevity, the remaining lifespan of an individual is defined as the time required for the withdrawal of the difference between the limiting value of alterations in the Individual Pangenome that is not compatible with life and the value of already altered DNA in the Individual Pangenome at a real-time point.

The model is based on characteristics that determine future system behavior and the rate of accumulation of alterations in the Individual Pangenome when considering the impact of external and intrinsic factors, in addition to the increased, progressive rate of alterations in the Individual Pangenome as a result of the accumulation of these alterations when one mutation induces another. External factors represent the totality of environmental exposure of different chemical (e.g., air pollution, pesticides, tobacco smoke, metals, and dietary factors), physical (e.g., UV radiation), and biological factors (e.g., infections) (Crew and Neugut 2006; Graf et al. 2015; Perera 1997; Schwabe and Jobin 2013). Intrinsic factors reflect intrinsic mutations that are acquired during normal cell division and include alterations that are caused by free radicals, along with those that are continuously produced during normal cellular respiration.

Based on Tetz’s theory of longevity, it is possible to compute the remaining lifespan defined as functions *q*_1_(*t*) and *q*_2_(*t*) depend upon time *t.* Functions *q*1(*t*) and *q*2(*t*) may be defined by ordinary differential equations:

9 $$\begin{aligned} & \frac{{{\text{d}}q_{1} }}{{{\text{d}}t}} = \alpha_{11} q_{1} + \alpha_{12} q_{2} + \sum\limits_{i = 1}^{N} {\beta_{1,i} } + \sum\limits_{f = 1}^{X} {\omega_{1,f} } , \\ & \frac{{{\text{d}}q_{2} }}{{{\text{d}}t}} = \alpha_{21} q_{1} + \alpha_{22} q_{2} + \sum\limits_{i = 1}^{N} {\beta_{2,i} } + \sum\limits_{f = 1}^{X} {\omega_{2,f} } . \\ \end{aligned}$$

Here, the left-hand-side contains the terms $\frac{{{\text{d}}q_{1} }}{{{\text{d}}t}}$ and $\frac{{{\text{d}}q_{2} }}{{{\text{d}}t}}$ that each provides the rate of alteration of DNA in macroorganism, microbiota, and their NGLEs at a real-time point. The right-hand-side contains terms that describe how different factors influence the rate of accumulation of alterations in the Individual Pangenome and life expectancy reduction:

- $\alpha_{11} = \sum\nolimits_{k = 1}^{l} {\alpha_{11,k}}$—increase in the rate of alterations in DNA of the macroorganism and associated NGLEs, due to the accumulation of alterations in the DNA of the macroorganism and associated NGLEs.
- $\alpha_{12} = \sum\nolimits_{k = 1}^{l} {\alpha_{12,k}}$—increase in the rate of alterations in DNA of the macroorganism and associated NGLEs, due to the accumulation of alterations in microbiota and associated NGLEs.
- $\alpha_{21} = \sum\nolimits_{k = 1}^{l} {\alpha_{21,k}}$—increase in the rate of alterations in DNA of microbiota and associated NGLEs, due to the accumulation of alterations in the DNA of the macroorganism and associated NGLEs.
- $\alpha_{22} = \sum\nolimits_{k = 1}^{l} {\alpha_{22,k}}$—increase in the rate of alterations in DNA of microbiota and associated NGLEs, due to the accumulation of alterations in microbiota and associated NGLEs.
- $\sum\nolimits_{i = 1}^{N} {\beta_{1,i}}$—impact of environmental factors on the incidence of alterations in the DNA of the macroorganism and associated NGLEs.
- $\sum\nolimits_{f = 1}^{X} {\omega_{1,f}}$—impact of intrinsic factors on the incidence of alterations in the DNA of the macroorganism and associated NGLEs.
- $\sum\nolimits_{i = 1}^{N} {\beta_{2,i}}$—impact of environmental factors on the incidence of alterations in the microbiome and associated NGLEs.
- $\sum\nolimits_{f = 1}^{X} {\omega_{2,f}}$—impact of intrinsic factors on the incidence of alterations in the microbiome and associated NGLEs.

The initial value problems are:

$$\begin{aligned} & q_{1} \left( {t_{0} } \right) = q_{1}^{\text{begin}} \\ & q_{2} \left( {t_{0} } \right) = q_{2}^{\text{begin}} . \\ \end{aligned}$$

**Fig. 1:**
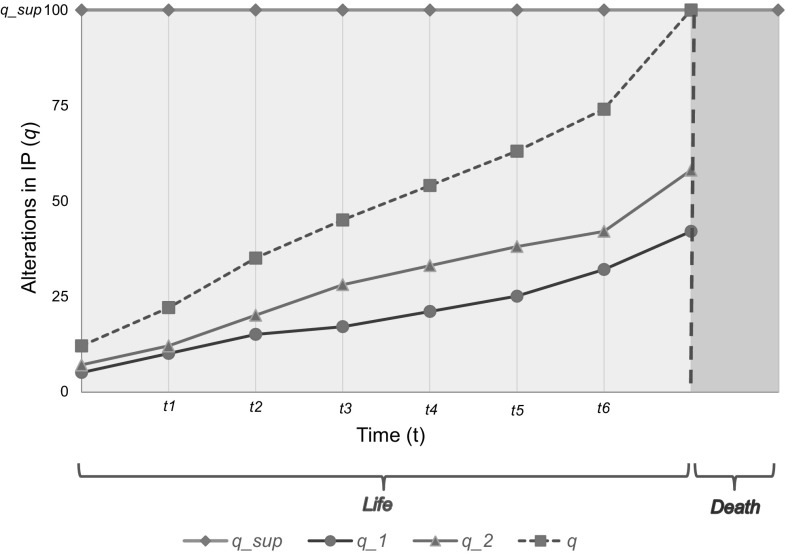
Solution of ordinary differential equations, showing the effect of *q*_1_ and *q*_2_ as a function of time (*t*, on the *X*-axis). The graph was produced using Eq. (10) that reflects the remaining lifespan. The lines were plotted using unspecified data to reflect the patterns of *q*, *q*1, and *q*2 as functions of time. The values on the *Y* axis represent the maximum number of alterations comparable with life, where *q*_sup = 100. The values on the *X*-axis represent time of alterations comparable with life in the interval [*t*0, *t*_sup]. The green zone and green brackets reflect the number of alterations in the Individual Pangenome that are compatible with life. The red zone and red brackets represent alterations that are not compatible with life, reaching the limiting value *q*^sup^ at *t*^sup^. In Fig. 1, *q* crosses the line *q*^sup^ at a certain *t*^sup^

The solution of ordinary differential equations produced a graph where *q*(*t*) =* q*_1_(*t*) + *q*_2_(*t*) (Fig. 1).

Then, at every time point *t*, the difference between *t*^sup^ and *t* reflects the remaining lifespan *t*^life^, whereby:

10 $$t^{\sup } {-}t = t^{\text{life}} .$$

Therefore, the model presented here derived from Tetz’s theory of longevity allows key traits of longevity to be determined based on the simultaneous effects of alterations in the DNA of the macroorganism, microbiota, and their NLGEs over a given lifespan.

## Discussion

Modeling the longevity of human life is the focus of much research (Medawar 1952; Hamilton 1966; Weinert and Timiras 2003). The Tetz’s theory of longevity proposed in this paper provides new explanations on the fundamental issues of species longevity and aging. This theory describes the longevity of the macroorganism using a supra-organismal approach (Eq. 1), termed the Individual Pangenome. This approach accounts for simultaneous genetic changes in the macroorganism, microbiota, and their NLGEs, leading to the death of the host. Tetz’s theory states that longevity and aging are defined by the accumulation of alterations in the total DNA of the host macroorganism, microbiota, and their NLGEs (Eq. 2), thus taking into consideration alterations that occur in both coding and non-coding regions of DNA (Buldyrev et al. 1995).

Based on Tetz’s theory, we also proposed Tetz’s law, stating that longevity is limited by the accumulation of alterations in the Individual Pangenome to the limiting value that is not compatible with life (Eqs. 3, 4). This limiting value of alterations may be determined for each species. Therefore, we state that longevity is defined as the time required for the accumulation of alterations in the Individual Pangenome to this limiting value. Alternatively, conceptually, the “life circle**”** is the withdrawal of the maximum capacity of alterations in the Individual Pangenome that are compatible with life. Our model allows alterations in the Individual Pangenome to be evaluated together with a broad variety of stochastic effects, including the outer environment (caused by physical, chemical, and biological agents), intrinsic mutations acquired during normal cell division, and an increased rate of alteration in the Individual Pangenome as a result of the accumulation of alterations that induce and trigger new ones (i.e., one alteration potentiates another). It is assumed that no qualitative or quantitative alterations in the Individual Pangenome occur without leaving a trace, thereby having a negative impact on the longevity of the host, including humans. This approach differs from other currently available approaches, which suggest that only around 4000 genes in humans have been associated with human health (Kellis et al. 2014). Tetz’s theory of longevity is based on the assumption that the host organism is immortal when there is ideal cooperation with microbiota, and environmental factors and intrinsic alterations are absent. This assumption allows us to take alterations in the Individual Pangenome into account, which are required for the growth of the organism, including age-dependent changes in the composition of microbiota (O’Toole and Claesson 2010). Therefore, it can be concluded that an organism could become immortal if, at any time point in an organism’s lifespan, the accumulation of alterations in the Individual Pangenome is blocked before reaching the limiting values of *q*^sup^.

Data based on the model developed here may have different levels of confidence depending on the accuracy of the input data of limiting value alterations in the Individual Pangenome and the accuracy of evaluation of the value of the existing alterations in the Individual Pangenome. To use the model developed here with the most accurate values that predict the remaining lifespan and speed of aging, it is necessary to advance and improve the existing methods of molecular-genetic and microbiological analysis on alterations in the composition of DNA (including genes) of macroorganism and microbiota. Although aging is a major focus of current medical research, the molecular mechanisms underlying longevity are only beginning to be elucidated. Today, there is a well-characterized normal range for the majority of mammalian genes (including humans); however, methods for the dynamic control (required for the highest accuracy of model) of alterations in their qualitative and quantitative totality are needed (The 1000 Genomes Project Consortium 2010; Sender et al. 2016).

However, the microbiome is much more poorly explored. According to different data, today, less than 20% of human commensal bacteria are characterized (Siqueira and Rocas 2013). Most bacteria, archaea, fungi, and protozoa of human microbiota cannot be cultivated at present, while molecular techniques based on cloning and sequencing the ribosomal 16S RNA are prone to false negatives, such as when one bacterial species masks another; consequently, bacterial diversity is underestimated (Li et al. 2012). Our theory also takes into account alterations that occur in NLGEs (viruses [including bacteriophages], plasmids, transposons, and extracellular DNA and RNA), which are important components of the Individual Pangenome. The composition of NLGEs in both the host and microbiome has received limited research, despite possessing broad multisystemic effects on the host organism. Such effects include the transfer of antibiotic resistance and the promotion of metastasis, as well as the direct lysis of certain eukaryotic and bacterial populations (Aarthy et al. 2015; Abeles and Pride 2014; Doolittle et al. 1995; Muniesa et al. 2013; Taylor et al. 2015). Yet, our proposed mathematical model provides a significant flexibility, with the input model parameters being easily tuned, so that the simulated data fit the actual scientific data well regarding the composition of macroorganism’ DNA and the microbiome. The model has high flexibility, because it accounts for both the quantitative and qualitative values of changes that are reflected in *q*, which, in turn, improves its accuracy, even without total knowledge of the Individual Pangenome and normal range of all the parameters. The qualitative values of the significance of impact of different alterations on host longevity are set manually, and can be adapted to match the most current level of knowledge (for example, including our knowledge of the difference about coding and non-coding DNA regions) or in accordance with a specific goal of model usage.

We used Tetz’s theory of longevity to develop a mathematical model that allows us to analyze and re-examine components that affect longevity. The remaining lifespan *t*^life^ is defined by solving ordinary differential equations for functions that show the effect of alterations in the Individual Pangenome as a function of time (Eqs. 9, 10). To compute the remaining lifespan, *t*^life^, the model takes into account a broad variety of stochastic effects, including the impact of environmental and internal factors. The manual input of these data will customize the significance of the role of specific factors. Thus, the role of different factors on the Individual Pangenome may be estimated, which achieves personalization of the characteristics that determine future alterations in the Individual Pangenome, depending on individual exposure to harmful factors, such as environmental, ecological, and dietary factors.

Another primary input factor of the model is age *q*^begin^. Our proposed model is valid for an every age, because it accounts for the level of preceding alterations, with the rate of alterations in the Individual Pangenome increasing over time, owing to the accumulation of alterations in the DNA of the macroorganism, microbiota, and their NLGEs. We suggest that the rate of alterations in the Individual Pangenome progressively increases as a result of the accumulation of these alterations, because one alteration induces/triggers another. The progressive nature of mutations when one alteration promotes another is well documented, and can be applied for an Individual Pangenome (Loeb et al. 2003).

The theory developed here allows the rate of aging (or, in other words, the rate of accumulation of alterations in the Individual Pangenome, i.e., the rate of *q*^max^ reduction) to be estimated, when assuming that aging and the rate of reduction of the remainder of the maximum permissible level of alterations are the same issues (Eqs. 5–8). Using Tetz’s theory of longevity and demographic comparisons between populations, it is possible to reveal Normal criterion on the rate of aging (i.e., rate of *q*^max^ reduction) at different age points. The estimation of this value represents an important personalized parameter that allows the predicted individual life expectancy to be evaluated based on the existing rate of aging, along with the influence of factors listed in Eq. 9. The accumulation of alterations in the Individual Pangenome is a dynamic process. In other words, we suggest that alterations due to intrinsic or environmental factors do not accumulate at a constant rate within and between individuals. The proposed model allows the control of the appearance and disappearance of alterations over time, which reflects the excess of the maximum permissible level of alterations in the Individual Pangenome over a certain time period. However, the model accounts for both primary alterations that may disappear within the time period and secondary alterations that are caused by the appearance of the primary ones, using the Δ*q* function. This situation is revealed in the “hit-and-run” theory that reflects the hypothesis that various agents initiate alterations in microbiota or host organisms, and then disappear leaving a cascade of pathological events and a dysfunctional immune system (Scarisbrick and Rodriguez 2003). Thus, using a number of assumptions and primary input factors that correspond to today’s knowledge on this field of research, Tetz’s theory of longevity incorporates both long-standing methodological and technical research issues with a relatively high level of confidence, despite methodological knowledge and instruments remaining imperfect for studying the Individual Pangenome.

## Conclusions

We suggest that the main goal of Tetz’s theory of longevity is to raise the upper limit on life expectancy. The proposed theory and mathematical model are the first to summarize how changes in the DNA of the macroorganism, microbiota, and their NLGEs have interdependent and inseparable effects on host longevity. Tetz’s theory of longevity possesses several implementations in medicine, allowing the influence of any disease and particularities of pathology to be evaluated in relation to organism lifespan. The proposed model is expected to provide a new outlook on the efficacy of the existing medications and medical procedures, by providing a way to quantitatively and qualitatively measure their impact on alterations of the Individual Pangenome and the rate of *q*(*t*) and *q*^max^ reduction.

In other words, the proposed model allows the efficacy of medical help to be re-examined, along with the consequences of diseases, from the perspective of how they affect the incidence and accumulation of alterations in the Individual Pangenome and, thus, how they affect life expectancy. This theory is also expected to inspire studies on the causes and particularities of aging, ways of evaluating how to correct and slow down aging, and how to prevent the aging process. The proposed theory assumes that human longevity is only limited by the limiting value of alterations in the Individual Pangenome. Thus, our future anti-aging research should focus on studying the concept of the Individual Pangenome, the rate of reduction of the remainder of maximum permissible level of alterations, ways to control this rate, and methods to achieve rate reduction.

Overall, Tetz’s theory of longevity provides explanatory and predictive potential on longevity and aging, allowing new fundamental aspects that control these processes to be examined. Tetz’s theory and law also provides a useful a tool for studying longevity, allowing the re-examination of the aging process and causes of mortality, as well as providing insights that may help to reduce rate of aging and extend human lifespans.
